# Comparison of the umbilical cord Blood’s anti-Mullerian hormone level in the newborns of mothers with polycystic ovary syndrome (PCOS) and healthy mothers

**DOI:** 10.1186/s13048-019-0583-4

**Published:** 2019-11-17

**Authors:** Faezeh Tadaion Far, Shahideh Jahanian Sadatmahalleh, Saeideh Ziaei, Anoshirvan Kazemnejad

**Affiliations:** 1 Department of Reproductive Health and Midwifery, Faculty of Medical Sciences, Tarbiat Modares University, Tehran, Iran; 2 Department of Biostatistics, Faculty of Medical Sciences, Tarbiat Modares University, Tehran, Iran

**Keywords:** Anti-Mullerian hormone (AMH), Polycystic ovary syndrome (PCOS), Ovarian function, Testicular function

## Abstract

**Background:**

Polycystic ovary syndrome (PCOS) is one of the most common endocrine diseases. At present, the cause of the disease is not fully understood, but many studies have shown that PCOS is associated with genetic and environmental factors. The present study aimed to assess the umbilical cord blood’s Anti-mullerian hormone (AMH) level in the newborns of mothers suffering from PCOS comparing to healthy mothers.

**Methods:**

This prospective cohort study was conducted on 120 pregnant women with PCOS, who were selected through Rotterdam criteria, and 60 healthy pregnant women as the control group. The subjects in each group were divided into obese and non-obese mothers according to their body mass index (BMI) before pregnancy. The cord blood samples were collected from the offsprings on the time of childbirth. Finally, the circulating concentrations of AMH in both sexes of the newborns were determined by specific assays.

**Results:**

The research results showed that the blood level of AMH was higher in the neonates of obese mothers with PCOS comparing to the controls (*P* < 0.001). Mean AMH level was higher in male neonates born from non-obese PCOS mothers than in the controls (P < 0.001); however, there was not a significant difference in the level of AMH in female neonates between these two groups (*P* = 0.264). Also the level of the above biomarker was higher in both sexes of the neonates belonging to obese PCOS mothers compared with the neonates born from non-obese PCOS mothers (*P* < 0.001).

**Conclusion(s):**

It can be said that the cord blood AMH level of neonates from obese women with PCOS is higher than that in the newborns of non-obese PCOS mothers. Further longitudinal studies are needed to confirm the clinical findings of the present research.

## Background

As the main cause of anovulatory infertility and hyperandrogenism, polycystic ovary syndrome (PCOS) is one of the most common (5–10%) endocrine-metabolic dysfunctions in women. There is altered folliculogenesis observed in this syndrome, meaning an excessive number of growing follicles comparing to normal ovaries [1, 2]. Anti-Mullerian hormone (AMH) is a dimeric glycoprotein member of the transforming growth factor-β (TGFβ) superfamily [3] that constitutes the marker of follicular development. It is exclusively produced in the gonads [4], whose dimorphic expression is critical in the testis and the ovary for the reproductive structures’ normal differentiation [5, 6]. During the early fetal development, the expression of Sertoli cells secreting AMH may end with Mullerian duct regression in men [4], whereas in the female fetus, the expression of granulosa cells secreting AMH takes place in the late gestation when the Mullerian ducts lose sensitivity to AMH [7]. Serum level of AMH seems to be associated with the development of preantral and small antral follicles, from puberty to the end of reproductive life [8]. During this period, the expression of AMH in the human ovary is much like to that in mice and rats [9], implying the crucial role of AMH in early follicle growth regulation. Research findings suggest that AMH level is 2–3 times higher in PCOS women than in healthy women during the reproductive life [1, 10]. Increase of the serum level of AMH in women with PCOS is associated with increase in the number of growing follicles secreting AMH [11]. Yet, it is to be known if AMH level increases before the clinical onset of PCOS, as defined by the Rotterdam ESHRE/ASRM-sponsored PCOS consensus [12]. Phenotypic and family aggregation investigations on different populations have shown that hyperandrogenic symptoms and polycystic ovaries are more frequent in the first-degree relatives of PCOS patients than in healthy controls [13–16]. The present study aimed to assess the umbilical cord blood’s AMH level in the newborns of mothers suffering from PCOS comparing to healthy mothers.

## Methods

This prospective cohort study was conducted aiming to assess the umbilical cord blood’s AMH level in the newborns of mothers suffering from PCOS comparing to the controls in Tehran City/capital of Iran.

The sample size was calculated based on the previous study [ 17] [mean and standard deviation in each group of PCOS and non-PCOS]. By considering a significance level of α = 0.05, power of 1-β = 0.80, and maximum sample size for this study was calculated. The sample size was determined at least 60 participants for each group.

The research population included 180 mother-newborn pairs consisting of 120 neonates from PCOS mothers, and 60 neonates from healthy mothers referring to an obstetrics clinic (for prenatal care) and to Arash Comprehensive Maternity Hospital affiliated to Tehran Medical Science University (Tehran, Iran).

The inclusion criteria were: spontaneous singleton pregnancy, the age range of 18–40 years, having the least literacy of reading and writing, and Iranian nationality.

The National Institute of Health’s (NIH) criteria (including chronic oligomenorrhea or amenorrhea and hirsutism or serum testosterone concentration of > 0.6 ng/ml and/or free androgen index (FAI) of > 5.0, and androstenedione concentration of > 3.0 ng/ml) were the bases for PCOS diagnosis in the present study [18].

The exclusion criteria were: smokers, history of chemotherapy or radiotherapy history, late-onset 21-hydroxylase deficiency, undergoing in vitro fertility, having hyperprolactinemia or androgen-secreting neoplasms, adult-onset congenital adrenal hyperplasia, the Cushing’s syndrome, and thyroid disease.

Healthy women with regular menstrual cycles were selected as the controls. They had no history of any chronic medication use, hirsutism, hyperandrogenism, gestational diabetes, galactorrhea, thyroid dysfunction, and hypertension. The two groups (control and PCOS) were matched in the mother’s age, education, employment, social-economical status, parity, and gestational age at labor, as well as the neonate’s gender.

Neonates with genetic disorders, malformation or preterm delivery were excluded from the study.

After obtaining their written consent, the subjects in both groups were divided into obese and non-obese mothers according to their body mass index (BMI) before pregnancy. In each PCOS group, 31 male and 29 female infants were assessed. As the control group, we studied 34 male and 26 female infants born from healthy mothers. The newborns in both groups (control and PSCO) enjoyed normal birth weight.

This study was conducted with the approval of the Institutional Review Board (IRB), and of Tarbiat Modares University’s Ethics Committee (IR.REC.1395.371). All the women were informed about the aims of the project and gave written consent before participating in the study.

### Laboratory measurements

The umbilical cord blood samples were collected immediately post-delivery, centrifuged and then frozen at − 45 °C. Serum AMH concentration of each umbilical cord blood sample was calculated using ELISA (Ct# A79765 Immunotech-France) with 0.7 picomole sensitivity, having dispersion coefficients 12.3 and 14.2% in intra-testing and inter-testing, respectively.

### Statistical analysis

The SPSS software (ver. 18.0) (SPSS Inc., Chicago, IL., USA) was employed for data analysis. In order to check the quantitative data, the statistical Kolmogorov-Smirnov’s test was applied (*P* < 0.05). Due to the abnormal distribution of the quantitative data of the research, Mann-Whitney’s U and Kruskal-Wallis H tests were used to find out the relationship between the variables (P < 0.05). Statistical significance was set at *P* < 0.05.

## Results

Totally, 180 pregnant women were assessed. In Table 1, the frequency distribution, and the mean and standard deviation(M ± SD) of the variables regarding the homogeneity of the groups are given. According to the study design, there was not a significant difference between the groups.

**Table 1:** Frequency distribution, the mean and standard deviation of the variables in the study population

Variables		Obese mothers with PCOS N = 60	Non-obese mothers with PCOS N = 60	Control *N* = 60	*P*-value
Mother’s age (months)* mean ± SD		28.51 ± 5.33	26.56 ± 4.39	28.06 ± 5.6	**0.076**
Parity (mean ± SD)*		0.7 ± 0.74	0.53 ± 0.72	0.78 ± 0.76	**0.117**
Gestational age at labor (weeks)*mean ± SD		37.81 ± 0.52	37.78 ± 0.52	37.81 ± 0.52	**0.944**
Economic status** (number/percent)	Weak	15(25)	18(30)	19(31.7)	**0.054**
	Normal	27(45)	28(46.7)	36(60)	
	Excellent	18(30)	14(23.3)	5(8.3)	
Neonate’s gender** (number/percent)	Male	31(51.7)	31(51.7)	34(56.7)	**0.818**
	Female	29(48.3)	29(48.3)	26(43.3)	
Employment status**(number/percent)	Employee	0	0	2(3.3)	**0.132**
	Housewife	60(100)	60(100)	58(96.7)	
Education** (number/percent)	Diploma	52(86.7)	52(86.7)	50(83.3)	**0.712**
	Academic	8(13.3)	8(13.3)	10(16.7)	

*Based on Kruskal-Wallis H test results **Based on Chi-square test results

As shown in Table 2, the Kruskal-Wallis test revealed a significant statistical difference in terms of the mean AMH levels between the subdivisions of the cases, as well as between the cases and the control group (*P* < 0.001).

**Table 2:** Anti-Mullerian hormone concentration in the cord blood of newborns in the study population

AMH (ng/ml) 푛푔푚푙)				
Gender Group	Male	95% CI	Female	95% CI
Obese cases having PCOS N = 60	27.07 ± 3.26	(25.87–28.26)	0.94 ± 0.31	(0.82–1.06)
Non-obese cases having PCOS *N* = 60	24.86 ± 1.89	(24.16–25.55)	0.34 ± 0.23	(0.25–0.43)
Non-obese control *N* = 30	23.15 ± 1.74	(22.25–24.04)	0.23 ± 0.12	(0.16–0.31)
Obese control N = 30	23.30 ± 0.63	(22.97–23.63)	0.25 ± 0.11	(0.18–0.32)
P-value	< 0.001		< 0.001	

Values are Mean ± SD (based on Kruskal-Wallis H test results)

AMH: Anti-Mullerian Hormone

CI: Confidence Intervals

The results indicated that the mean AMH levels in the cord blood of newborns in both sexes were statistically higher in the obese mothers suffering from PCOS (male: 27.07 ± 3.26 vs. female: 0.94 ± 0.31) as compared to the controls (male: 23.30 ± 0.63 vs. female: 0.25 ± 0.11, *P* < 0.001). Mean serum level of AMH in the cord blood of the male neonates of non-obese PCOS mothers was statistically higher than in the controls (24.86 ± 1.89 vs. 23.15 ± 1.74, *P* < 0.001). However, the comparison was not statistically significant in case of the female newborns (0.34 ± 0.23 vs. 0.23 ± 0.12, *P* = 0.264). Also the mean AMH levels in the cord blood of newborns in both genders born from obese mothers with PCOS (male: 27.07 ± 3.26 vs. female: 0.94 ± 0.31) compared with the non-obese PCOS mothers (male: 24.86 ± 1.89 vs. 0.34 ± 0.23) were significantly higher (*P* < 0.001), but there was no significant difference between the mean level of AMH in the cord blood of male (23.30 ± 0.63) and female (0.25 ± 0.11) infants from obese control mothers with male (23.15 ± 1.74) and female (0.23 ± 0.12) infants from non-obese control mothers (*P* = 0.522, *P* = 0.871) (data not shown).

## Discussion

The current study aimed at assessing the umbilical cord blood’s AMH level in the newborns of PCOS mothers comparing to healthy control mothers.

It was found that the mean serum AMH concentration of the umbilical cord blood of the neonates born from obese/non-obese PCOS mothers was higher as compared to the controls (*P* < 0.001). However, regarding the female neonates, in spite of the considerable difference between the serum AMH level in the two groups (non-obese mothers with PCOS/controls), no significant statistical difference was observed between them (*P* = 0.264). Also the mean level of AMH was higher in the cord blood of neonates of obese mothers with PCOS comparing to the non-obese PCOS women (*P* < 0.001).

In the following, some of the studies done on PCOS are presented:

In 2006, Teresa-Sir Petermann et al. compared 2–3 months old and 4–7 years old prepubertal girl infants of mothers with PCOS and those born from healthy mothers (control group). The results showed that the serum level of AMH in the PCOS group was significantly higher than in the control group during the infancy and childhood [19].

Nicolas Crisosto et al. (2007) compared the daughters (8–16 yr old) born from PCOS and healthy women. The results indicated that the PCOS group had significantly higher AMH level than their control counterparts [20].

In 2008, Sergio E. Recabarren et al. [21] compared the males (infants, children, and adults) born from PCOS and healthy mothers. It was revealed that the PCOS group had higher AMH level than the controls during both infancy and childhood.

In 2011, Nicolas Crisosto et al. found that AMH serum level in the female neonates born from mothers with PCOS, who had not been treated with metformin during pregnancy, was higher compared to the control group; however, its level in the female neonates born from mothers having PCOS, who had been treated with metformin during pregnancy, was similar to that in the control group [17].

In 2012, Teresa –Sir Petermann et al. reported that the AMH serum level and the ovarian volume in the case group were more than those in the control group during all Tanner stages [22]. But we evaluated the serum concentrations of AMH in the cord blood of both sexes of newborns from PCOS and healthy mothers.

The above studies confirm our findings. Though we assessed the umbilical cord blood’s AMH level in the newborns of PCOS mothers to the controls. It was found that AMH concentration of the umbilical cord blood of the neonates from obese/ nonobese PCOS mothers was higher as compared to the the controls. This phenomenon can be justified with regard to environmental or genetic factors. PCOS has a genetic etiology [23], as well as many candidate genes [24]. In addition, the endocrine, nutritional, and metabolic milieu of the fetus may have a programming effect, which may persist in adult life [25–27]. Further more, PCOS women show a considerable increase in androgen level during pregnancy, as a potential androgen source to the fetus [28]. Hence, possibly, high levels of androgen observed in pregnant women suffering from PCOS may affect the fetal physiology and thus lead to the ovarian morphology and function changes as described in the experimental models. Research findings on rhesus monkeys imply that intraovarian androgens inhibit apoptosis and promote granulosa cell proliferation, especially in small follicles having granulosa cells rich in androgen receptors [29]. In a recent experimental study, male sheep born to testosterone-exposed mothers exhibited an increased FSH receptor expression in the testis [21]. Under the condition of androgenic inhibition, FSH increases the serum level of AMH through promoting the proliferation of Sertoli cells [30]. Thus, it can be said that the serum level of AMH increases in the offspring of PCOS women can be indicative of an increase in the ovarian follicular reserve quantity or hyperactivity of Sertoli cells. Besides, contrary to other studies, the current study findings do not confirm a significant relationship in the cord blood AMH levels of female neonates from non-obese mothers suffering from PCOS and the controls. This can be due to more sample size of the above studies.

Also, based on the results of some previous studies [31–34], the strong independent positive effect of Luteinizing Hormone (LH) on the granulosa cell production of AMH has been demonstrated in the consistently higher AMH levels in normal-weight women, comparing to obese and overweight women. Although in a previous report on adolescent PCOS girls [34], no significant association was observed between AMH levels and BMI. In two other studies [35, 36], AMH levels were lower by 65% in the obese women than in the normal-weight women of late reproductive age. Therefore, the serum level of AMH has a reverse relationship with BMI in individuals suffering from PCOS. But the present study indicated opposite results to previous suggestions in this rgard. This is justifiable under the hypothesis that often the women with PCOS have circumstantial resistance to insulin, which plays a key role in the pathogenesis of the syndrome. Eventually, hyperinsulinemia is linked to hyperandrogenism, since insulin acts in the theca cells, potentiating the effects of LH on steroidogenesis. Insulin also has a stimulating effect on steroidogenesis in the granulosa cells, increasing androgen levels and resulting in a greater number of follicles in growth starting from the follicle recruitment phase, suggesting a correlation with increased AMH production [37]. A recent study on the effects of Myo-inositol on serum AMH in patients with PCOS showed a significant improvement in AMH, fasting glucose, ovarian volume, ovarian antral follicle, and total antral follicle count [38]. It may improve clinical and hormonal features of PCOS patients by enhancing insulin sensitivity that decreases [39]. There is evidence that the occurrence of insulin resistance in non-pregnant women suffering from PCOS varies between 50 and 80% [37]. In addition, obesity, through reducing insulin sensitivity and increasing the availability of glucose for maternal-fetal transport [40], promotes intrauterine growth [41]. Moreover, it has been demonstrated that obese women exhibit higher glucose levels during pregnancy than normal-weight women. Even if these glucose levels do not reach the range for gestational diabetes, the impact on the fetal pancreas is similar to that of gestational diabetes [42] so that it causes fetal hyperinsulinemia. Besides, insulin – as a trophic factor for granulosa and Sertoli cells- may increase the number of AMH producing cells although we cannot exclude the possibility that each granulosa and Sertoli cell could produce more AMH in response to insulin. Conversely, a higher number of follicles reflected by high AMH levels could determine higher androgen levels related to hyperinsulinemia [43]. AMH is found may increase to three to four-fold in PCOS patients as evidenced by previous studies. Elevated AMH concentration in PCOS patients is largely due to increased production by individual follicles rather than increased follicle number [44], which may confound its association with ovarian reserve and/or quality, thus explaining the poor predictability of AMH. Furthermore, available data suggest the role of AMH in inhibiting folliculogenesis by interference with the concentration and the actions of FSH in the ovaries [45]. some previous studies [46, 47] reported a reduced ovarian response in PCOS women when AMH level is above a threshold value (4.7 ng/ml). Therefore, PCOS patients require dose step up during ART cycles, and are at higher risk for severe OHSS.

The present research, had some limitations. First, the sample size is small and the findings are inconsistent with the results of some other works, may be due to this reason. Second, we did not divide the patients with PCOS into four groups based on Rotterdam criteria. Piouka et al. [36] reported the different levels of AMH in four groups.

The strength of the current study was that the differences observed could not be explained by some confounding factors because some parameters were matched as far as possible between the four groups. Also the increased levels of AMH in umbilical cord blood could reflect the endocrinal effect of motherʾs hormone on the earlier developement of the fetus.

## Conclusions

It is concluded that the cord blood AMH level of neonates from obese women with PCOS is higher than in the newborns of non-obese PCOS mothers. In order to reach a conclusion with more certainty, it is recommended to do a comparative study with large scope of PCOS mothers with male and female newborns.

## Abbreviations

AMH: Anti-Mullerian Hormone
BMI: Body Mass Index
FAI: Free Androgen Index
IRB: Institutional Review Board
LH: Luteinizing Hormone
NIH: National Institute of Health
PCOS: Polycystic Ovary Syndrome
TGFβ: Transforming Growth Factor-β
